# Detection of Crimean-Congo hemorrhagic fever virus in blood-fed *Hyalomma* ticks collected from Mauritanian livestock

**DOI:** 10.1186/s13071-021-04819-x

**Published:** 2021-06-29

**Authors:** A. Schulz, Y. Barry, F. Stoek, M. J. Pickin, A. Ba, L. Chitimia-Dobler, M. L. Haki, B. A. Doumbia, A. Eisenbarth, A. Diambar, M. Y. Bah, M. Eiden, M. H. Groschup

**Affiliations:** 1 Institute of Novel and Emerging Infectious Diseases, Friedrich-Loeffler-Institut, Südufer 10, 17493 Greifswald-Insel Riems, Germany; 2 Office National de Recherches et de Développement de l’Elevage (ONARDEL), Nouakchott, Mauritania; 3 Bundeswehr Institute of Microbiology, Neuherbergstrasse 11, 80937 Munich, Germany; 4 Ministère du Développement Rural, Nouakchott, Mauritania

**Keywords:** Crimean-Congo hemorrhagic fever virus, *Hyalomma* species, Livestock, Epidemiology, Mauritania

## Abstract

**Background:**

Crimean-Congo hemorrhagic fever virus (CCHFV) belongs to the genus *Orthonairovirus* (*Nairovididae*) and is a (re)emerging tick-borne pathogen. It is endemic in most parts of Africa, Asia and southern Europe, and can cause severe hemorrhagic symptoms in humans, with high fatality rates (5–30%).

**Methods:**

*Hyalomma* ticks were collected from four different livestock herds (cattle and camels) in Mauritania in 2018. The tick species were determined morphologically and confirmed molecularly by using the cytochrome oxidase 1 gene marker. For the detection of CCHFV, ticks were tested individually by one-step multiplex real-time reverse-transcriptase quantitative polymerase chain reaction. The small segment of all positive samples was sequenced to determine the CCHFV genotype.

**Results:**

In total, 39 of the 1523 ticks (2.56%) collected from 63 cattles and 28 camels tested positive for CCHFV. Three *Hyalomma* species were identified. *Hyalomma rufipes* had the largest proportion of positivity (5.67%; 16/282), followed by *Hyalomma dromedarii* (1.89%; 23/1214). No *Hyalomma impeltatum* tested positive (0%; 0/21). Positive ticks were found in only six out of 91 host animals. Viral sequence analysis revealed the presence of two different CCHFV lineages (Africa I and Africa III).

**Conclusions:**

In this study, 2.56% of *Hyalomma* ticks collected from camels and cattle in Mauritania tested positive for CCHFV. However, the true prevalence of CCHFV in unfed ticks may be lower, as a considerable number of ticks may have been passively infected during blood-feeding by co-feeding ticks or due to viremia of the host. The results indicate the need to track the actual area of circulation of this virus.

**Graphic Abstract:**

**Supplementary Information:**

The online version contains supplementary material available at 10.1186/s13071-021-04819-x.

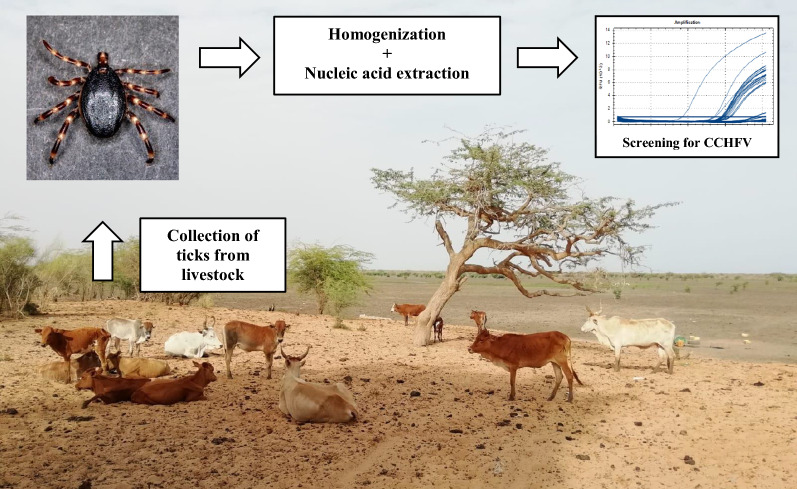

## Background

Crimean-Congo hemorrhagic fever virus (CCHFV), which is a member of the genus *Orthonairovirus* in the family *Nairovididae*, is an emerging zoonotic arthropod-borne virus that causes Crimean-Congo hemorrhagic fever (CCHF) in humans. CCHFV is present in most parts of Africa, southern Asia and southern Europe [1, 2]. The virus is characterized by its high genetic diversity [3]. Hard ticks of the genus *Hyalomma* are considered to be the main vectors and reservoirs of CCHFV [4]. Most, if not all, of the various hosts of *Hyalomma* (ranging from wildlife species to domesticated animals) can be infected with CCHFV, although they tend to develop only short-term viremia without clinical symptoms [5]. In contrast, CCHFV infections in humans can lead to severe hemorrhagic symptoms, with a high lethality rate of up to 30% [4]. Seroepidemiological studies of different livestock and wildlife species can provide a first indication of whether CCHFV is circulating in a region. Hence seroepidemiology has become a widely used instrument to identify areas potentially at risk of endemicity of the virus [5, 6]. For direct virus detection in these risk areas, ticks collected in the course of field studies are often screened for the presence of the virus. However, these findings must be interpreted with caution. The detection of CCHFV in engorged ticks is only evidence of the presence of the virus—it does not imply vector competence of a tick, or constitute a quantitative measure of the true virus prevalence in an area, since passive contamination of the tick from the blood of the host cannot be excluded [7]. Unequivocal information on host-vector dynamics as well as vector competence can only be obtained by complex experimental studies of tick-host transmission, which have to be conducted according to stringent guidelines, as described by Gargili et al. [7]. According to World Health Organization data, West African countries, including Mauritania, are considered highly endemic, with a large annual incidence of human CCHF cases. Traditional husbandry is common in Mauritania, meaning that there is close contact between farmers and their livestock. The first evidence of human CCHFV infection in Mauritania was reported in 1983 [8]. Moreover, several serological studies on livestock revealed a high seroprevalence, 15%, in small ruminants [9, 10] and up to 67% in cattle [11], underlining the high endemic status of the country. Despite those high seroprevalences, the presence of CCHFV in ticks has not been systematically studied in Mauritania. The first comprehensive survey was conducted in 1985 and included 2539 ticks collected from cattle, sheep, goats, camels and horses [12]. The samples were pooled and analyzed using the complement fixation test, which showed 12/172 (6–9%) CCHFV-positive tick pools. Of the four analyzed tick species (*Hyalomma rufipes*, *Hyalomma marginatum*, *Hyalomma impeltatum* and *Hyalomma dromedarii*), only *H. rufipes* pools were positive. In a second study, 378 engorged and non-engorged ticks of two different genera (*Hyalomma* and *Rhipicephalus*) were tested for the presence of CCHFV. CCHFV was only detected in four *Rhipicephalus evertsi evertsi* collected from sheep, whereas all of the *Hyalomma* ticks were negative [10].

The main goal of this study was to obtain recent data on the circulation of CCHFV in selected livestock herds and *Hyalomma* ticks in Mauritania. Special attention was paid to the characterization of CCHFV genotypes and accurate tick species determination, since incorrect identification of tick species is considered to be one of the primary reasons for misleading statements regarding potential vector/reservoir species in the literature [7, 13]. The results presented here can contribute to a better CCHFV exposure risk analysis for local farmers, butchers and other exposed groups in close contact with livestock in the surveyed sampling areas. However, this work is not intended to replace the large-scale, nationwide epidemiological studies that are necessary for a comprehensive and detailed risk analysis.

## Methods

### Collection sites

**Fig. 1:**
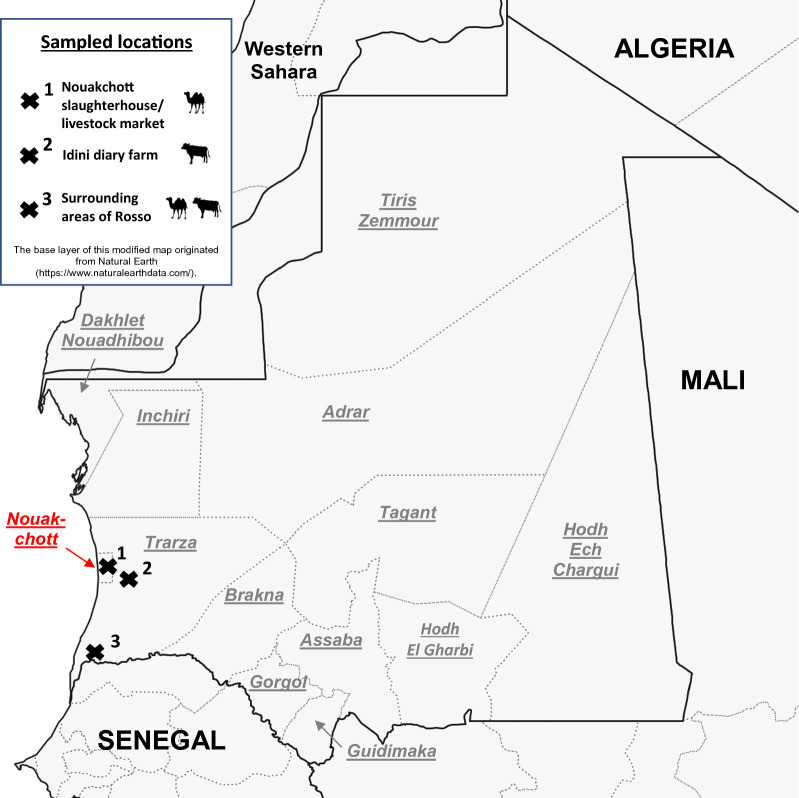
Map of Mauritania and its neighboring countries showing the different sampling sites for cattle and camels

Mauritania, a large country in West Africa, is mainly located in the desert landscape of the Sahara, and has a low population density. Samples were collected in the region surrounding the capital Nouakchott and the town of Rosso. Camels were sampled at a livestock market with a nearby slaughterhouse on the outskirts of Nouakchott. In addition, one cattle herd was sampled at a dairy farm 60 km east of Nouakchott near the small village Idini (Trarza). Two other sampling sites for cattle and camels, respectively, were located in the area surrounding Rosso (Trarza) in southwestern Mauritania. The distance between Nouakchott and Rosso is about 160 km (Fig. 1).

### Collection of samples

*Hyalomma* ticks were collected from a total of 28 camels and 63 cattles from the four aforementioned herds. On average, about 17 ticks per animal were collected and examined. The highest number of ticks (53 specimens) from an individual animal were collected from a cattle in Idini, while the lowest number (two specimens) was collected from a cattle in Rosso. Blood samples were taken from most of the animals (Idini, cattle, *n* = 49; Nouakchott slaughterhouse, camels, *n* = 13; Rosso, camels, *n* = 15). No blood samples were obtained from the cattle herd in Rosso. In total, 77 blood samples were collected among the different herds. The sampled animals were mainly infested with ticks of the genus *Hyalomma*. Given that *Hyalomma* ticks are considered to be the main vector of CCHFV and that the primary objective was to detect as well as to characterize the virus, ticks of other genera were not considered in this study. The collected ticks and sera were stored at − 70 °C for 5 months at the Office National de Recherche et de Développement de l'Elevage in Nouakchott. Ethanol (90%) was added to the tick samples prior to their shipment to the Friedrich-Loeffler-Institut, Germany. Subsequently, tick and blood samples were stored in ethanol for 3 months before further processing.

### Morphological and molecular identification of tick species

All ticks were morphologically identified using the identification keys of Apanaskevich et al. [14–16]. Individual ticks were homogenized in Buffer AVL (300 µl AVL buffer plus one 5-mm steel bead; Qiagen, Hilden, Germany) in a TissueLyser II (Qiagen, Hilden, Germany). The homogenates were cleared by centrifugation and the supernatants were used for nucleic acid extraction. DNA/RNA was extracted using a KingFisher Flex (ThermoFisher, Waltham, USA) with the NucleoMag VET kit (Macherey–Nagel, Düren, Germany) according to the manufacturer’s protocol. A selected number of ticks that were hard to determine, as well as CCHFV-positive specimens, were identified using partial cytochrome oxidase subunit 1 gene (*CO1*) Sanger sequencing and restriction fragment length polymorphism [17].

### CCHFV genome detection

RNA extracted using a KingFisher Flex (ThermoFisher) with the NucleoMag VET kit (Machery-Nagel) from individual ticks and serum samples was used to screen samples for CCHFV. The screening was performed using a one-step real-time reverse-transcriptase polymerase chain reaction assay (RT-qPCR) as described previously [18]. The assay targets a conserved region within the small (S) segment. Samples were considered positive where cycle threshold (Ct) values were below 35 and weakly positive if between 35 and 40.

### Molecular and phylogenetic analyses of CCHFV genotypes

To obtain initial information on the detected CCHFV genotypes, amplicons (127 bp) of the RT-qPCR products of all positive samples were sequenced by Sanger sequencing (Eurofins Scientific, Luxembourg, Luxembourg) and aligned with GenBank entries by using the BLAST tool (National Center for Biotechnology Information, Bethesda, MD). For this purpose, the PCR protocol was performed using only one primer pair specific to the African CCHFV lineage III. Due to the short length of the PCR product, it was necessary to amplify a larger part of the S segment, which allows for a more meaningful phylogenetic analysis. Therefore, complimentary primers close to the terminal regions were selected based on the most related sequence in the BLAST results. The primers used to amplify African linage 1 were forward 5’-AACACGTGCCGCTTACGC and reverse 5′–TATCGTTGCCGCACAGCC, and for African linage 3, forward 5′–ATGGAAAACAAAATCGAGGTGAATAACAAAGAT and reverse 5′–TTAGATAATGTTAGCACTGGTGGCATT. The reverse transcription using SuperScript IV Reverse Transcriptase (ThermoFisher) with the reverse primer and the PCR using the KAPA HiFi HotStart ReadyMix PCR kit (Roche, Basel, Switzerland) were performed according to the manufacturers’ instructions. The amplified fragment was sequenced by Sanger sequencing (Eurofins Scientific) and used to create a phylogenetic tree.

### Statistical analysis

Statistical analyses included 95% confidence intervals (CI), Fisher's exact test and χ^2^-test. R software and RStudio (an integrated development interface for R) were used for the calculations [19]. Those ticks that could not be identified to species level were excluded from the Fisher exact test and χ^2^-test.

## Results

### Tick species and their distribution on hosts

Overall, 1523 blood-fed *Hyalomma* ticks were collected from 63 cattle and 28 camels. Morphological identification revealed the presence of three different tick species (*H. dromedarii*, *H. rufipes* and *H. impeltatum*) in the surveyed areas. Due to the large morphological diversity of *Hyalomma* ticks and the similarity of *H. dromedarii* and *H. impeltatum*, 219 specimens were identified genetically by the restriction fragment length polymorphism approach. Furthermore, the *CO1* gene amplicon used for the restriction digest was sequenced from 47 ticks to unambiguously identify them to species level. Due to the very poor condition of six ticks, neither their morphological nor molecular identification was possible. Therefore, they were only determined to genus level. These six specimens were not considered for CCHFV screening. The results of the species identifications and distribution among the different collection sites and hosts are summarized in Table 1. *H. dromedarii* was by far the most common tick species in both cattle and camels (80.03%), followed by *H. rufipes* (18.59%) and *H. impeltatum* (1.38%). Despite the predominance of *H. dromedarii*, variation in the regional distribution of tick species on their hosts was high. In both camel herds, the percentage of *H. dromedarii* was very high (97.01–98.58%), while ticks of the other species were found only sporadically. In contrast, the distribution of tick species among the two cattle herds was considerably more heterogeneous. A large number of *H. dromedarii* (89.65%) were identified on the dairy farm in Idini, although *H. rufipes* (7.01%) and *H. impeltatum* (3.34%) were also recorded there. However, 85.34% of the ticks collected from the cattle herd in Rosso were identified as *H. rufipes* and only 14.66% as *H. dromedarii*. The distribution of sex showed nearly a 70:30 ratio of males to females across all four collection sites (Table 2). In the cattle herd from Rosso, the proportion of male ticks was slightly higher compared to the other sampling sites.

**Table 1:** Crimean-Congo hemorrhagic fever virus (CCHFV) detection in ticks at the different sampling sites

Location	No. sampled animals	Tick species	No. ticks collected	%	No. of CCHFV-positive ticks	Pos./total (95% CI)	Pos./tick species (95% CI)
Idini	43 Cattles	*Hyalomma dromedarii*	537	89.65%	20	3.33% (2.05–5.12%)	3.72% (2.29–5.69%)
		*Hyalomma impeltatum*	20	3.34%	0	0% (0–0.06%)	0% (0–16.84%)
		*Hyalomma rufipes*	42	7.01%	9	1.5% (0.06–2.8%)	21.43% (10.3–36.81%)
		Total	599		29	4.84% (3.27–6.88%)	*p*-value < 0.001
Nouakchott slaughterhouse	14 Camels	*H. dromedarii*	346	98.58%	3	0.85% (0.78–2.48%)	0.87% (0.18–2.51%)
		*H. rufipes*	5	1.42%	0	0% (0–1.05%)	0% (0–52.18%)
		Total	351		3	0.85% (0.78–2.48%)	*p*-value (NA)^a^
Rosso	20 Camels	*H. dromedarii*	39	14.66%	0	0% (0–1.38%)	0% (0–9.03%)
		*H. rufipes*	227	85.34%	7	2.63% (1.06–5.35%)	3.08% (1.25–6.25%)
		Total	266		7	2.63% (1.06–5.35%)	*p*-value (NA)^a^
Rosso	14 Camels	*H. dromedarii*	292	97.01%	0	0% (0–1.21%)	0% (0–1.26%)
		*H. impeltatum*	1	0.33%	0	0% (0–1.21%)	0% (0–95.7%)
		*H. rufipes*	8	2.66%	0	0% (0–1.21%)	0% (0–36.94%)
		Total	301		0	0% (0–1.21%)	*p*-value 0.531
Total	91 cattle and camels	*H. dromedarii*	1,214	80.03%	23	1.51% (0.96–2.27%)	1.89% (0.96–2.26%)
		*H. impeltatum*	21	1.38%	0	0% (0–0.24%)	0% (0–16.11%)
		*H. rufipes*	282	18.59%	16	1.05% (0.6–1.71%)	5.67% (3.28–9.05%)
		Total	1517		39	2.56% (1.83–3.5%)	*p*-value < 0.001

Overview showing results for all four sampled herds, including the number of individuals of different tick species collected, CCHFV-positive ticks (*Pos*.), and analysis of the correlation between the three identified species and CCHFV status by χ^2^-test (*p* < 0.05)

*CI* Confidence interval, *NA* not available (due to the absence of *H. impeltatum* at the sampling site)

**Table 2:** Number of female and male sampled ticks and their CCHFV status

Location	Sex (tick)	*n*	Pos.	CCHFV pos./total (95% CI)	CCHFV pos./sex (95% CI)	*p*-value
Idini (cattle)	Female	188 (31.39%)	8	1.33% (0.58–2.61)	4.26% (1.85–8.13)	0.8378
	Male	411 (68.61%)	21	3.51% (2.18–5.31)	5.12% (3.19–7.7)	
Nouakchott slaughterhouse (camels)	Female	106 (30.20%)	2	0.57% (0.07–2.04)	1.89% (0.23–6.65)	0.2178
	Male	245 (69.80%)	1	0.28% (0–1.58)	0.41% (0.01–2.25)	
Rosso (camels)	Female	83 (27.57%)	0	0% (0–1.22)	0% (0–4.35)	1
	Male	218 (72.43%)	0	0% (0–1.22)	0% (0–1.68)	
Rosso (cattle)	Female	59 (22.18%)	0	0% (0–1.38)	0% (0–6.06)	0.354
	Male	207 (77.82%)	7	2.63% (1.37–6.84)	3.38% (1.37–6.84)	
Total (cattle and camels)	Female	436 (28.71%)	10	0.66% (0.32–1.21)	2.23% (1.11–4.18)	0.724
	Male	1081 (71.26%)	29	1.91% (1.28–2.73)	2.68% (1.8–3.83)	

Overview of the sex ratios of the ticks across the different collection sites and their CCHFV status. For abbreviations, see Table 1

### Molecular CCHFV diagnostics for ticks and sera

A comprehensive summary of the PCR results is given in Table 1. There were significant differences between tick species and between sampling sites in the number of CCHFV-positive ticks. The highest number of positive ticks (29/599; 4.84%) was found in the cattle herd from Idini (Trarza). Overall, in Idini, 20 of 537 *H. dromedarii* (3.72%) and nine of 42 *H. rufipes* (21.43%) were CCHFV positive, while all *H. impeltatum* ticks (*n* = 20) were negative. Only three *H. dromedarii* collected from camels at the Tenweich slaughterhouse tested positive (0.87%)*.* In contrast, all ticks from the camel herd near Rosso (Trarza) tested negative, whereas seven out of 266 ticks (all *H. rufipes*) originating from cattle of the same region tested positive (2.63%). In total, 2.23% of the female and 2.68% of the male ticks were CCHFV positive. No significant differences were observed in CCHFV status between male and female ticks (Table 2). Despite CCHFV-positive ticks being found, all 77 serum samples of camels and cattle were negative for CCHFV.

### Distribution of CCHFV-positive ticks per sampled animal

The distribution of all CCHFV-positive *Hyalomma* spp. collected on cattle and camels is summarized in Table 3. CCHFV-positive ticks were collected from six out of 91 (6.59%) sampled animals (five cattles, nos. 1–5, and one camel, no. 1). In one case (cattle no. 1), all 22 collected ticks were CCHFV positive. In three cattles (nos. 2–4), at least one tick tested positive (weakly positive). In each of four cattles (nos. 1–4), one of the collected ticks was highly positive, showing much higher levels of S segment RNA (a lower Ct value) compared to the other ticks collected from the same cattle. Three of these highly positive ticks were identified as *H. rufipes*; the other tick was identified as *H. dromedarii*. In cattle no. 5, only a single *H. rufipes* was CCHFV positive, while the remaining ticks were negative. In camels, only the ticks collected from camel no. 1 (*n* = 3; *H. dromedarii*) tested positive for CCHFV. Viral RNA concentrations of the S segment were almost identical between all of these three ticks, and no weakly positive ticks were found.

**Table 3:** Distribution of positive ticks amongst the sampled animals

Location	Host	*n*	+	(+)	Tick samples with the lowest CCHFV Ct value				CCHFV
					Lowest Ct value	Tick species (lowest Ct value)	Second-lowest Ct value	Tick species (second-lowest Ct value)	Genotype
Idini	Cattle no. 1	22	22	0	18.26	*H. rufipes*	28.62	*H. dromedarii*	Africa I
Idini	Cattle no. 2	30	1	8	19.44	*H. dromedarii*	35.22	*H. dromedarii*	Africa I
Idini	Cattle no. 3	22	5	9	19.68	*H. rufipes*	34.44	*H. dromedarii*	Africa I
Rosso	Cattle no. 4	21	7	3	28.8	*H. rufipes*	31.52	*H. rufipes*	Africa III
Idini	Cattle no. 5	8	1	0	26.66	*H. rufipes*	–	–	Africa III
Nouakchott	Camel no. 1	24	3	0	28.67	*H. dromedarii* (×3)	(29.89; 29.56)	–	Africa I
Total		127	39	20					

All sampled animals on which CCHFV-positive ticks were found (6/91), including weakly positive ticks, and the tick species of the most CCHFV-positive sample. The CCHFV genotype was determined by comparing the real-time reverse-transcriptase polymerase chain reaction (127 bp) data using the BLAST tool (National Center for Biotechnology Information). For abbreviations, see Table 1

*n*  Number of ticks, + positive ticks, (*+*) weakly positive ticks, ×3 (3 specimens of H. dromedarii)

### Phylogeny of CCHFV genotypes

**Fig. 2:**
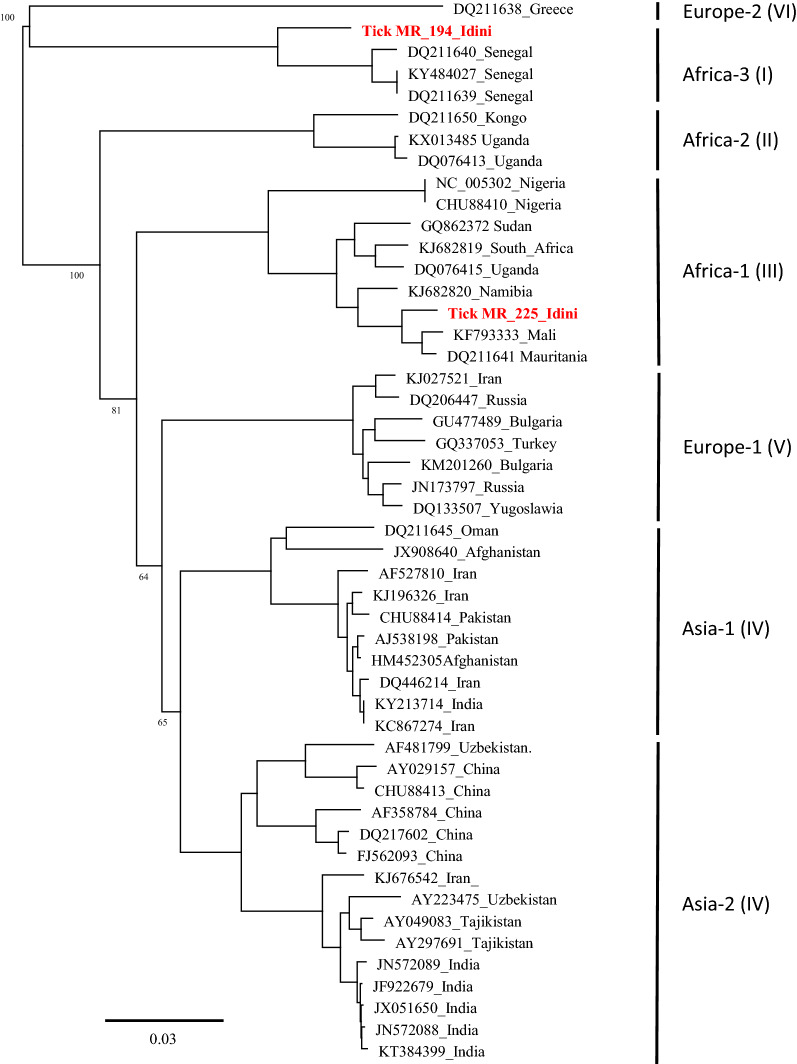
Phylogenetic tree showing the genetic distances of the small (*S*) segment of the consensus strains and selected Crimean-Congo hemorrhagic fever virus-positive samples from Mauritania (1384 bp). The tree was generated using the neighbor-joining algorithm and Jukes-Cantor distance model in Geneious version 2019.2 (Biomatters, available from https://www.geneious.com). The tree was midpoint rooted using FigTree v1.4.4 (available from https://github.com/rambaut/figtree/releases).* Values at nodes* represent support from 1,000 bootstrap replicates. Bootstrap values are only shown for major branches

To determine the CCHFV genotypes, the 127-bp-long RT-qPCR amplicons were sequenced. Consistent sequence data were obtained for 28 of the 39 CCHFV-positive ticks and compared by using the BLAST tool (National Center for Biotechnology Information). Essentially, two different CCHFV genotypes—Africa I and Africa III—were detected (Table 3; Additional file 1: Table S1). Ticks feeding on the same animal always carried the same CCHFV genotype and haplotype. However, two different lineages (Africa I and III) circulated simultaneously in ticks from the Idini region (Table 3). The CCHFV lineages also differed between the collection sites and/or host species (Additional file 1: Table S1). The Africa I lineage detected in ticks from cattle (Idini) and camels (Nouakchott slaughterhouse) showed nucleotide differences of 3.15%. The Africa III genotypes found in ticks feeding on cattle from Idini and from Rosso varied by 2.17%. The overall genetic distance between the Africa I and Africa III lineages ranged from 10.04% between the cattle herds from Idini (I) and Rosso (III) and up to a maximum of 13.19% between camels from Nouakchott slaughterhouse (I) and cattle from Rossi/Idini (III). To confirm that the short amplicon (127 bp) was representative of the whole segment, the complete coding region of the S segment from two CCHFV-positive ticks was RT-PCR amplified and sequenced. Alignments with GenBank database sequences showed a nucleotide homology of 97.5% and 99.4% for the consensus sequences of Africa I and Africa III strains, respectively. Construction of a phylogenetic tree (Fig. 2) showed that both sequences clustered well with the reference strains of Africa I (Senegal) and III (Mauritania/Mali).

## Discussion

Previous studies have shown a high CCHFV antibody seroprevalence in the livestock population of Mauritania, and several severe cases of CCHF have been reported in humans in the country. Hence, Mauritania is considered highly endemic for CCHFV [8–11, 20]. CCHF cases have also been reported in Senegal, which borders Mauritania to the south [21, 22]. In Mali, which borders Mauritania to the east, no cases of CCHF have been reported in humans, but serological data [23] and virus detection in ticks [24] have proven that CCHFV circulates there. CCHFV monitoring in ticks (especially of the genus *Hyalomma*) has not yet been conducted systematically in Mauritania. Existing datasets from most studies carried out in the West African region are either small, outdated or were generated by the analysis of tick pools with a focus on virus detection, and thus only allow limited conclusions to be drawn on tick species distributions [10, 12, 24, 25]. Therefore, the present study was carried out to provide a better understanding of the current epidemiology of CCHFV in selected Mauritanian livestock herds as well as in *Hyalomma* ticks themselves.

The presence of three tick species, *H. rufipes*, *H. dromedarii* and *H. impeltatum*, in Mauritania is consistent with previous findings in the region [14–16]. The primary hosts of *H. dromedarii* are, as its specific name suggests, camels [16], which explains the high proportion of tick specimens (97.01–98.58%) found on camels in Rosso and the Nouakchott slaughterhouse (Table 1). Due to their importance as livestock for milk and meat production in Mauritania, there is a relatively high camel density in the country [26]. Moreover, cattle and other ungulates can also be infested with adult stages of *H. dromedarii* [16], especially if camels and cattle are kept in close proximity. Thus, the fact that 89.65% of the *H. dromedarii* ticks were found on cattle from Idini was not considered an exceptional finding.

The highest number of ticks tested positive (Table 1) was found in the cattle herds from Idini (4.84%) and Rosso (2.63%), followed by the camels from the Nouakchott slaughterhouse (0.85%). No CCHFV was detected in ticks collected from camels in Rosso. The reasons for varying levels of CCHFV-positive ticks across collection sites and host species are manifold and require careful interpretation. It should be noted that only blood-fed ticks were examined in this study. Hence, these results only give an indication of the presence of CCHFV in ticks from the studied regions. Thus, the term “CCHFV prevalence” has not been used to describe the data of the examined ticks, as it remains unclear whether the ticks infected the host animals or vice versa. However, CCHFV was not detected in the sera. Nevertheless, some statistically significant deviations were observed between the collection sites for the respective tick species, which should not be ignored. One explanation for these deviations could be a difference in susceptibility between cattle and camels to the virus. So far, only a small number of experimental CCHFV infection studies have been conducted on different livestock species (cattle, sheep and horses); these studies showed that all of these species developed short-term viremia of similar duration [6]. However, no experimental data of CCHFV infections of camelids are presently available to support this suspected difference in susceptibility between cattle and camels. There remains a need for further infection experiments that compare the susceptibility of different host species to CCHFV.

The CCHFV status of a tick may also depend on the species. Ticks of the genus *Hyalomma* are considered to be the main vectors and reservoirs of CCHFV [7], but it is still unknown whether all of the currently recognized 27 *Hyalomma* spp. [27] are efficient virus reservoirs and/or vectors. Despite the considerably higher detection rate of CCHFV for *H. dromedarii* ticks (79.71%) in the study region (Table 1), significantly more *H. rufipes* (5.67%) than *H. dromedarii* ticks (1.89%) were CCHFV positive. This difference was most obvious among the cattle herd from Idini, where 21.43% of *H. rufipes* and only 3.72% of *H. dromedarii* were CCHFV positive. Nevertheless, since our data were derived from blood-fed ticks only, conclusions cannot be drawn on differences in vector competence of the different species. Furthermore, feeding on viremic hosts and/or co-feeding transmission [28–31] may also have contributed to the concentrated occurrence of some of the 39 positive ticks collected from six of 91 animals (Table 3). Interestingly, one of the CCHFV-positive ticks collected from four cattles in Idini and Rosso (nos. 1–4) showed a considerably lower Ct value than the co-infesting ticks from the same animals (Table 2). The four ticks collected from these cattle apparently harbored a very high viral load, and may have been responsible for co-feeding transmissions of the other infected ticks in addition to causing CCHFV infection in the host animals. It is also noteworthy that three of four highly positive ticks in Idini (total occurrence: 89.95% *H. dromedarii* vs. 7.01% *H. rufipes*) were identified as *H. rufipes* (Table 3), which may indicate that this tick species has a higher vector competence for CCHFV. The genomic data proving 100% sequence identity of CCHFV for all infected ticks (at least for the 28 sequenceable samples) from a given bovine host support this assumption (Table 3; Additional file 1: Table S1). The degree of blood-feeding may also affect the sensitivity of the PCR. This may explain the low Ct values especially in females, which can ingest many times their own weight in blood. However, all the specimens with a particularly low Ct value were males. Therefore, the true CCHFV prevalence in the unfed adult tick population may actually be considerably lower than that calculated (2.56%). There is also circulation of the virus in larval and nymphal stages, which also pose an exposure risk of an unknown scale. Nevertheless, enforcing tick control strategies and encouraging public awareness of tick bite prevention in the examined areas is recommended.

**Fig. 3a–d:**
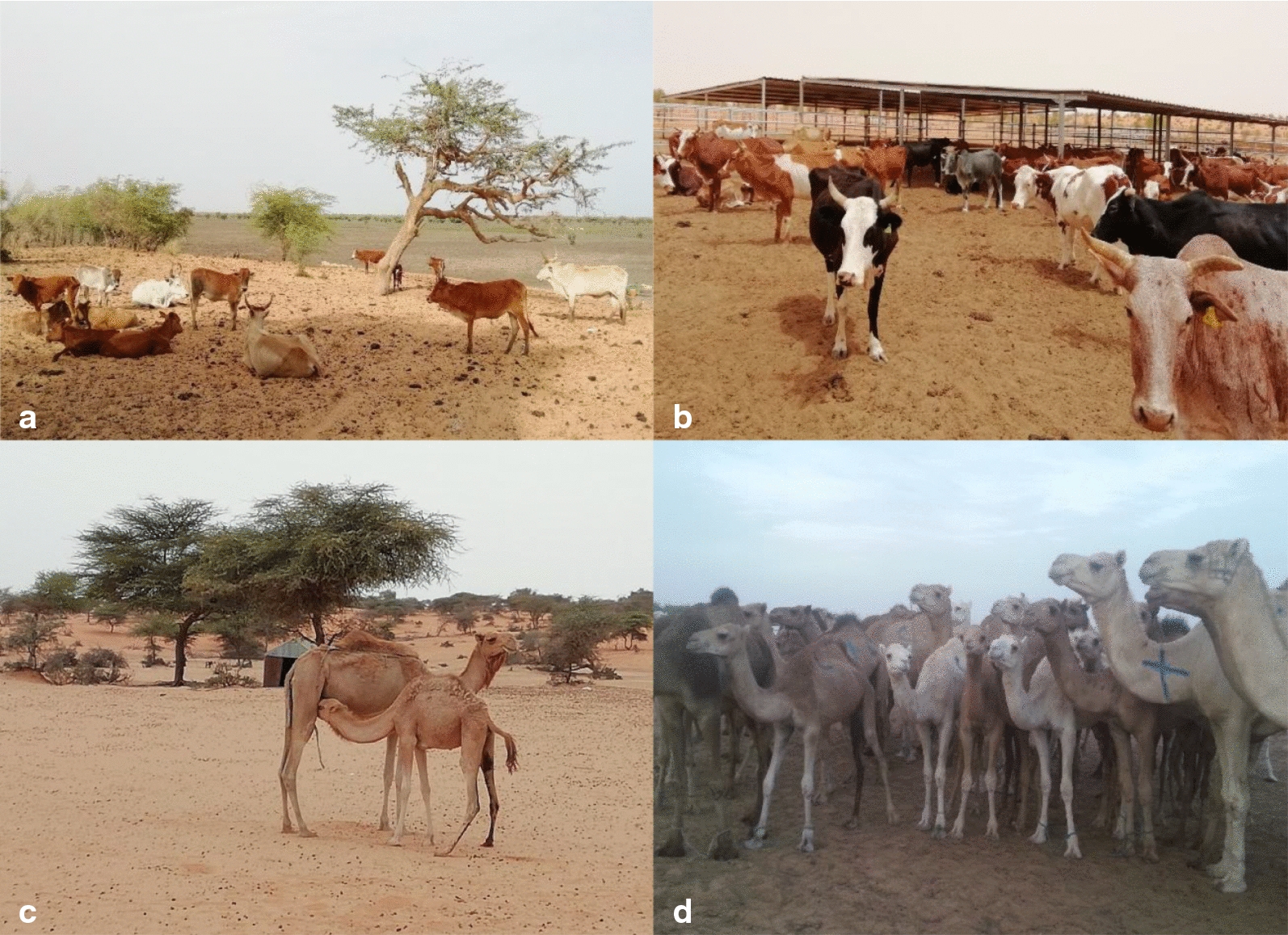
Different habitats of the livestock herds (cattle and camels) selected for sampling. Cattle (**a**) and camel herds (**c**) grazing near Rosso (Trarza) close to the Senegal river (southern Sahel). The favorable climatic conditions permit the growth of large trees in the savanna landscape and also the cultivation of crops. In contrast, a modern dairy farm in Idini (**b**) is completely surrounded by desert. **d** Camels gathered together for sale at the highly frequented Nouakchott livestock market/slaughterhouse

The high prevalence of CCHFV in Idini may be related to the isolated location of the sampled dairy farm. It is assumed that fragmented CCHFV foci consisting of susceptible hosts and competent vector ticks may induce stable virus amplification, leading to a high prevalence in isolated geographical clusters [32]. This dairy farm is located in a desert-like region far away from the village of Idini. Its remote location results in limited contact between the cattle and new naive hosts (wildlife, livestock), which might negatively affect the CCHFV prevalence [33]. In contrast, the fertile lands around Rosso have led to a higher density of livestock as well as a higher density of the human population, and thus to increased movement and interaction between the animals. A similar situation exists at the livestock market in Nouakchott, where a large number of cattle, sheep, goats and camels from various regions of Mauritania are brought for sale or slaughtered every day (Fig. 3). At least two different CCHFV genotypes (Africa I and III) were found in ticks in Mauritania, either alone or circulating together, as observed in one cattle herd from Idini (Table 3; Additional file 1: Table S1). Genetic variability also occurred within the genotypes. The underlying mechanisms of this high genetic diversity are still not fully understood and require further research to elucidate the driving factors.

## Conclusions

This study revealed the presence of CCHFV in *Hyalomma* ticks collected from camels and cattle in Mauritania. Significantly more *H. rufipes* than *H. dromedarii* and *H. impeltatum* tested positive for CCHFV. However, it should be taken into consideration that the data were obtained from engorged ticks, thus true prevalence or vector competence could not be determined. Two different CCHFV genotypes (Africa I and III) were found in the ticks. The absolute risk for local farmers of being bitten by unfed adult ticks is probably lower than the calculated prevalence would suggest, since a considerable number of ticks may have been passively infected during ingestion of the blood meal by co-feeding with infected ticks, or by feeding on a host that was already viremic. Nonetheless, the risk of CCHFV exposure through direct contact with viremic host blood, or when crushing ticks during their removal from animals, remains, as does the risk posed by questing larval and nymphal stages. Thus, enforcing tick control strategies and encouraging public awareness of tick bite prevention in these areas is recommended. The findings of this study point to the urgent need for large-scale epidemiological studies across Mauritania to achieve a comprehensive risk analysis of exposure to CCHFV in the country.

## Supplementary Information

**Additional file 1: Table S1.** Genetic distances (%) between the CCHFV genotypes.Genetic distances (%) between the CCHFV lineages found in the respective positive ticks on cattle and camels deduced by comparing the real-time reverse-transcriptase polymerase chain reaction amplicons (127 bp). In all positive ticks originating from the same host animal, both the genotype and the detected gene sequence were identical.

## Data Availability

All data generated or analyzed during this study are included in this published article.
