# Supplementary material for: Detection of Crimean-Congo hemorrhagic fever virus in blood-fed Hyalomma ticks collected from Mauritanian livestock
Source: Parasit Vectors. 2021 Jun 29;14:342. doi: 10.1186/s13071-021-04819-x (PMC8244218; doi:10.1186/s13071-021-04819-x)
Supplement: Supplementary file 1 — Additional file 1: Table S1. Genetic distances (%) between the CCHFV genotypes.Genetic distances (%) between the CCHFV lineages found in the respective positive ticks on cattle and camels deduced by comparing the real-time reverse-transcriptase polymerase chain reaction amplicons (127 bp). In all positive ticks originating from the same host animal, both the genotype and the detected gene sequence were identical. [file 13071_2021_4819_MOESM1_ESM.docx]

**Table S1. Genetic distances (%) between the CCHFV genotypes**

Genetic distances (%) between the CCHFV lineages found in the respective positive ticks on cattle and camels by comparing the RT-qPCR amplicons (127 bp). In all positive ticks originated from the same host animal, both the genotype and the detected gene sequence were identical.

| **Host/location/genotype** | | | **Cattle No.1** | **Cattle No.2** | **Cattle No.3** | **Cattle No.4** | **Cattle No.5** | **Camel No.1** |
| --- | --- | --- | --- | --- | --- | --- | --- | --- |
|  |  |  | Idini | Idini | Idini | Rosso | Idini | Nouakchott |
|  |  |  | Africa I | Africa I | Africa I | Africa III | Africa III | Africa I |
| **Cattle No.1** | Idini | Africa I | - | 100 % | 100 % | 89.96 % | 89.76 % | 96.85 % |
| **Cattle No.2** | Idini | Africa I | 100 % | - | 100 % | 89.96 % | 89.76 % | 96.85 % |
| **Cattle No.3** | Idini | Africa I | 100 % | 100 % | - | 89.96 % | 89.76 % | 96.85 % |
| **Cattle No.4** | Rosso | Africa III | 89.96 % | 89.96 % | 89.96 % | - | 97.83 % | 86.81 % |
| **Cattle No.5** | Idini | Africa III | 89.76 % | 89.76 % | 89.76 % | 97.83 % | - | 86.81 % |
| **Camel No.1** | Nouakchott | Africa I | 96.85 % | 96.85 % | 96.85 % | 86.81 % | 86.81 % | - |
